# microRNA-365 inhibits YAP through TLR4-mediated IRF3 phosphorylation and thereby alleviates gastric precancerous lesions

**DOI:** 10.1186/s12935-020-01578-0

**Published:** 2020-11-13

**Authors:** Tianqi Zhang, Kunpeng Zhang, Kaiyue Ji, Cuiping Zhang, Yueping Jiang, Qi Zhang, Zibin Tian, Xinyu Wang, Mengyuan Zhang, Xiaoyu Li

**Affiliations:** Department of Gastroenterology, The Affiliated Hospital of Qingdao University, No. 16, Jiangsu Road, Shinan District, Qingdao, 266000 Shandong People’s Republic of China

**Keywords:** MicroRNA-365, TLR4, IRF3, YAP, CDX2, Gastric precancerous lesions

## Abstract

**Background:**

Gastric carcinoma (GC) is currently one of the most common malignant tumors of the digestive system, and gastric precancerous lesions play a vital role in studying the mechanism of GC. Multiple microRNAs (miRNAs) have been documented to be potential biomarkers to indicate progression of gastric precancerous lesions. In this study, we explained the anti-cancer effect of miR-365 in gastric precancerous lesions via regulation of the TLR4/IRF3/YAP/CDX2 axis.

**Methods:**

miR-365, TLR4, CDX2 and IPF3 expression was determined in GC and atrophic gastritis tissues and cells. After transfection of shRNA and overexpression plasmids, in vitro experiments detected the alteration of cell viability, apoptosis and inflammatory factors. Bioinformatics analysis, Co-IP and dual luciferase reporter gene assay were conducted to evaluate the binding between miR-365 and TLR4 as well as IRF3 and YAP. Rat models were established to explore the effect of the miR-365 and TLR4 on gastric precancerous lesions.

**Results:**

miR-365 was poorly expressed in GC and atrophic gastritis tissues and GC cell lines, while TLR4, CDX2 and IRF3 were overexpressed. Of note, miR-365 was indicated to target TLR4 and thereby suppressed cancer progression and increased hemoglobin content. Interestingly, silencing of TLR4 was accompanied by decreased IRF3 phosphorylation and reduced expression with less binding between CDX2 and IRF3. Downregulation of YAP resulted in declined CDX2 expression in cancer cells. Moreover, the inhibitory role of miR-365 was further confirmed in animal models.

**Conclusion:**

Taken together, miR-365-mediated TLR4 inhibition reduces IRF3 phosphorylation and YAP-mediated CDX2 transcription to impede progression of gastric precancerous lesions.

## Background

Gastric cancer (GC), the most common gastrointestinal malignant tumor, is primarily characterized by anorexia, dyspepsia, weight loss and abdominal pain [1]. H pylori infection and other various factors such environmental factors induce the pathogenesis of GC [2]. However, despite improvements in surgery and chemotherapy for GC, the 5-year overall survival rate is still unsatisfactory [3]. GC is usually accompanied by gastric precancerous lesions that are more prone to become cancerous in pathology. Accumulating evidence has demonstrated the role of microRNAs (miRNAs) in GC, such as miR-144 [4], miR-532 [5], miR-494 [6], miR-449c-5p [3], miR-129-3p [7]. In human GC tissues, miR-365 reduction correlates with poorly differentiated histology, deep invasion and advanced stage [8]. In addition, miR-365 was predicted to target TLR4 and inhibit its expression by in silico analysis in the present study.

Meanwhile, existing data have demonstrated the involvement of TLR4 in GC where the expression of TLR4 is closely related to the TNM stage and lymph node metastasis of GC [9]. It has been reported in the literature that TLR4 promotes the occurrence of cancer by regulating the expression of the downstream protein interferon regulatory factor 3 (IRF3) [10, 11]. IRF3 is a well-defined signal transduction factor/transcription factor that is essential for the innate antiviral response. IRF3 promotes the nuclear translocation and activation of YAP by interacting with YAP and TEAD4 in the nucleus, thereby promoting the occurrence of GC [12, 13]. Existing research indicates that Wnt3a may activate and regulate CDX2 expression through the WNT-YAP/TAZ signaling pathway, and thus play a key role in the maintenance of bovine TSC [14]. DNA methylation was partly responsible for CDX2 silencing in GC [15] and CDX2 also reduced the migration and invasion of GC cells [16]. On the basis of the aforementioned information, we conducted a cascade of in vitro and in vivo assays based on the miR-365/TLR4/IRF3/YAP/CDX2 axis to identify novel biomarkers involved in GC tumor progression in order to improve the prognosis and further understand the exact molecular mechanism of GC.

## Methods

### Ethics statement

The current study was performed with the approval of the ethics committee of the Affiliated Hospital of Qingdao University. All participants signed informed consent documentation prior to sample collection. The animal experimental processes were approved by the Ethnic Committee of the Affiliated Hospital of Qingdao University and conducted in strict accordance to *the Guide for the Care and Use of Laboratory Animals* published by the US National Institutes of Health.

### Clinical sample collection

The study subjects consisted of 68 patients with GC hospitalized at the Affiliated Hospital of Qingdao University from April 2016 to September 2018, and 45 patients with atrophic gastritis. Their age was 32–73 years, with a mean age of 45.6 years, including 38 males and 30 females. The GC tissue and adjacent normal tissues (more than 5 cm from the tumor edge) were collected, and the surgically resected specimens were immediately stored in liquid nitrogen. All specimens were confirmed by pathological examination, and patients received no radiotherapy or chemotherapy before surgery.

### *Tumor necrosis factor-alpha (TNF-α)-induced immortalized normal gastric mucosal epithelial cell line *in vitro

Human gastric mucosal epithelial cells (GES-1) (C0355, ATCC, USA) were cultured in a high-glucose DMEM containing 10% FBS at 37 °C under 5% CO_2_ at constant temperature and humidity overnight. When cells reached 80–90% confluence, 0.3 mL of TNF-α was added to the culture well to induce GES-1 into gastric EMT-transformed model cells with a malignant transformation tendency, and the control group was added with an equal amount of distilled water.

### shRNA screening

TLR4 gene sequence was retrieved in GenBank database, and shRNAs of TLR4 that specifically knocks down TLR4 gene fragments were designed (Table 1) and constructed into the pshRNA-Neo plasmid. The successfully constructed TLR4 gene silencing plasmid following enzyme digestion and sequencing was named sh-TLR4-1 and sh-TLR4-2. TLR4-shRNA vector and negative control vector were transfected into GSE-1 cells. RT-qPCR was employed to detect the content of TLR4 in cells to screen the most effective shRNA sequence.

**Table 1:** shRNA and negative control sequences

Gene	Sequence (5′-3′)
sh-TLR4-1	5′-CACGGCATCTTTACTGGCTTAGTCA-3′
sh-TLR4-2	5′-CATCTTCACAGAGCTGACTAACTTA-3′
negative control	5′-TTCTCCGAACGTGTCACGTTT-3′

### Cell culture and grouping

Human normal gastric mucosal epithelial cell line RGM-1 (GDC214350849-03, Guandao Biotech. Shanghai, China) and GC cell line MGC-803 (3111C0001CCC000227, Cell Resource Center, Institute of Basic Medical Research, Chinese Academy of Medical Sciences), MKN-45 (3111C0001CCC000229, Cell Resource Center, Institute of Basic Medical Sciences, Chinese Academy of Medical Sciences), and HGC-27 (3111C0001CCC000279, Cell Resource Center, Institute of Basic Medical Sciences, Chinese Academy of Medical Sciences) were cultured in basic medium containing 8% DMSO and 20% FBS (12633012, Haoran Bio, Shanghai, China) in a 5% CO_2_ incubator at 37 °C. The cells at logarithmic growth phase were tested. The induced GES-1 cells were transfected with plasmids of NC mimic, miR-365 mimic, NC inhibitor + sh-NC, miR-365 inhibitor + sh-NC, miR-365 inhibitor + sh-TLR4, sh-NC, sh-TLR4, oe-NC, oe-TLR4, sh-NC + oe-NC group, sh-TLR4 + oe-NC and sh-TLR4 + oe-CDX2. The above plasmids were purchased from Dharmacon (Lafayette, CO, USA).

### Animal model establishment

Wistar rats weighing 180–200 g were selected for establishing a rat model of GC related to chronic gastritis using helicobacter pylori suspension (50 μL, approximately10^6^ CFU) and drinking water containing 100 mg/mL *N*-methyl*N*-nitro-*N*-nitroso-guanidine (MNNG). GC model was established after fed for 4 to 6 months combined with MNNG. The process from gastric precancerous lesions to GC was simulated, during which the lesions in the stomach was observed and pathological intestinal metaplasia (atypical hyperplasia) and canceration were confirmed. The rats were then treated with NC agomir, miR-365 agomir, NC antagomir + sh-NC, miR-365 antagomir + sh-NC, miR-365 antagomir + sh-TLR4, sh-NC + oe-NC, sh-TLR4 + oe-NC and sh-TLR4 + oe-CDX2.

### ELISA

The bottom of a kit (rat, ab208113, Abcam, USA; human NeoBioscience, EHC007.48, Shenzhen) was coated with specific IL-6 antibodies. The plasma and specific biotinylated IL-6 antibody combined with the IL-6 in the sample were added to the kit and incubated at room temperature, after which unbound biotinylated antibody was washed away and streptavidin-peroxidase conjugate was added. After the reaction, unbound conjugates were washed away. The tetramethylbenzidine (TMB) contained in color development solution can be catalyzed by streptavidin peroxidase to produce a blue conjugate, which turned yellow after the addition of acid stop solution. The density of the yellow conjugate was proportional to the IL-6 content in the sample at the bottom of the kit. The optical density (OD) value of the yellow solution was measured by a microplate reader, and a standard curve was drawn to calculate the IL6 content in the sample.

IL-1 content measurement was the same as above using IL-1 kit (rat, ab9722, Abcam, USA; human Cayman, 583311-96, Beijing, China).

### Cell counting kit-8 (CCK-8) assay

The cells were seeded into a 96-well plate at a density of 2 × 10^3^ cells/well. A blank control group containing only medium and no cells was set for zeroing. After 24 h transfection, CCK-8 solution (10 μL) was added to each well at 0, 24, 48, 72 and 96 h, and incubated for an additional 4 h at 37 °C. The absorbance at 450 nm was measured using a microplate reader (Bio-Rad, Hercules, CA, USA). The ratio of absorbance value in the experimental group to that in the control group was calculated and a cell viability curve was plotted.

### Flow cytometry

On the second day after transfection, the cells of each group were detached with 0.25% trypsin which was terminated with RPMI-1640 medium containing 10% fetal bovine serum. Following after, the cells were centrifuged at 1000 r/min for 5 min, with the supernatant discarded, fixed with 70% pre-chilled ethanol to a concentration of 1 × 10^6^ cells/mL, and then stained with 10 mL of Annexin V-FITC/PI (556547, Shanghai Shuojia Biotechnology Co., Ltd.) for 15–30 min in a refrigerator at 4 °C. Cell apoptosis was measured using flow cytometer (XL type, Conter Company, USA). Fluorescence was initiated by excitation at 488 nm (FITC) and 530 nm (FITC) and was measured at more than 575 nm (PI). The apoptosis rate was analyzed using flow cytometry software SYSTEM IIV 3.0 and presented as the percentage of the number of apoptotic cells in total number of cells.

### Hematoxylin eosin (HE) staining

The rat gastric tissue was extracted, fixed, then embedded in paraffin conventionally, and cut into 4 µm sections. The sections were then dewaxed with xylene (xylene I for 5 min and xylene II for 5 min), and rehydrated in ascending series of alcohol (100% alcohol for 2 min, 95% alcohol for 1 min, 80% alcohol for 1 min, 75% alcohol for 1 min), washed with distilled water for 2 min and stained with hematoxylin for 5 min and rinsed under tap water. The sections were hydrolyzed for 30 s with hydrochloric acid ethanol, soaked in tap water for 15 min or 50 °C water bath for 5 min, and then stained with eosin for 2 min, followed by conventional dehydration, clearing, and mounting. Finally, the results were analyzed under an inverted microscope (XSP-8CA, Shanghai Optical Instrument Factory, Shanghai, China).

### Plasma analysis

Blood sample was collected from the abdominal aorta of rats, and 6 mL of blood sample was collected into an EP tube, which was added with heparin for anticoagulation. The sample was centrifuged for 15 min and the plasma viscosity was measured after 2 h using a R-80A automatic flushing blood viscosity tester (Beijing Shidi Scientific Instrument Co., Ltd., Beijing, China). Another 2 mL of blood was collected with a biochemical tube and anticoagulated with potassium EDTA. Next, the amount of hemoglobin was measured with a fully automatic blood cell analyzer (Beijing Shidi Scientific Instrument Company, Beijing, China).

### Gastric juice analysis

Gastric juice was diluted 50 times with 0.04 moL/L hydrochloric acid solution, after which 0.5 mL of diluted gastric juice was taken out and mixed with 2 mL of hemoglobin matrix solution at 37 °C and allowed to stand for 10 min. Afterwards, 5% trichloroacetamide (5 mL) was added, mixed and centrifuged, followed by addition of 5 mL of sodium carbonate solution. The absorbance at 640 nm was measured with a 721-type spectrophotometer (Shanghai Optical Instrument, Shanghai, China) to determine pepsin activity.

### Nuclear-cytoplasmic fractionation assay

The cells were washed with Buffer A, then centrifuged at 500*g* for 5 min, lysed with BufferA + B (2: 1), mixed gently, and incubated for 5–10 min, followed by another centrifugation at 12,000*g* for 10 min (supernatant: cytoplasm; pellet: nucleus and cell membranes). The cells were washed twice with PBS, centrifuged at 12,000*g* for 10 min. Afterwards, Buffer C was employed to lyse cell membrane (the cells were quickly frozen in liquid nitrogen and thawed on ice for 10 min) which was centrifuged at 12,000*g* for 10 min (supernatant: cell nucleus; pellet: cell membrane) and washed with PBS. Additionally, the cells were treated with PRPA buffer on ice for 30 min, and centrifuged at 12,000*g* for 10 min (supernatant: cell membrane). Subsequently, Western blot analysis was employed to detect the the extent of IRF3 phosphorylation with Histone H3 employed as the internal protein reference for nuclear protein and GAPDH as the internal reference for cytoplasmic protein.

### Dual-luciferase reporter assay

Bioinformatics was employed to analyze the binding relationship between miR-365 and TLR4, which was verified through dual-luciferase reporter assay. The binding sites between miR-365 and TLR4 were mutated, and TLR4 mutant plasmid was constructed for dual-luciferase reporter experiments. The artificially synthesized TLR4-3′UTR gene fragment was introduced into pMIR-reporter (Beijing Huayueyang Biotechnology Co., Ltd., Beijing, China) using the endonuclease sites SpeI and Hind III, and a complementary sequence mutation site of the seed sequence was designed on the TLR4 wild type (WT). After restriction endonuclease, T4DNA ligase was employed to insert the target fragment into the pMIR-reporter plasmid. The correctly sequenced luciferase reporter plasmids TLR4 WT and TLR4 MUT were co-transfected with miR-365 into HEK-293T cells (Shanghai Institutes for Biological Sciences, Chinese Academy of Sciences, Shanghai, China). 48 h after transfection, cells were collected and lysed. The luciferase activity was detected with the luciferase detection kit (K801-200, Biovision Technologies, USA) and a Glomax20/20 luminometer fluorescence detector (Promega Corporation, USA). Three values of each sample were recorded: RLU1, firefly luciferase activity; RLU2, renilla luciferase activity, and RLU1/RLU2, the ratio of firefly and renilla activity.

### Co-IP assay

Cells were lysed with RIPA lysis buffer and added with 1% cocktail (1: 100, sigma). After sonication, cells were lysed on ice at 4 °C, and then cell debris was removed by centrifugation. The cell lysate was incubated with 1 μg of antibody to IRF3 (1: 1000, ab68481, Abcam, UK), IgG (1: 2000, ab6721, Abcam UK), and 15 μL of protein A/G beads (Santa Cruz Biotechnology) for 2 h. After extensive washing, the beads were boiled at 100 °C for 5 min. Proteins were separated by sodium dodecyl sulfate-polyacrylamide gel electrophoresis (SDS-PAGE), transferred to a nitrocellulose membrane (Millipore, Temecula, CA, USA), and then immunoblotted.

### Immunohistochemistry

Tissue sections were heated in a 60 °C incubator for 1 h, dewaxed with xylene conventionally, hydrated with gradient ethanol, and incubated with 0.5% Triton in PBS at room temperature for 20 min. After antigen retrival under high pressure for 2 min, the sections were heated in 0.01 M citrate buffer (pH 6.0) at 95 °C for 20 min, and immersed in 3% H_2_O_2_ for 15 min to block exogenous peroxidase activity. Then the sections were blocked with 3% BSA blocking solution and incubated with the diluted primary antibodies: rabbit anti-mouse TLR4 (1: 500, ab13556, Abcam, UK) or rabbit anti-mouse CDX2 (1: 10,000, ab76541, Abcam, UK) at 37 °C for 2 h, followed by washing with PBS. Afterwards, the sections were then added with HRP-labeled goat anti-rabbit IgG secondary antibody working solution (ab6721, 1: 1000, Abcam, Cambridge, UK) for incubation in a humidified box at 37 °C for 30 min. After incubation, tissue sections were counterstained with hematoxylin (Shanghai Fusheng Industrial Co., Ltd., Shanghai, China) at room temperature for 4 min, and the excess stain was rinsed under running water. Finally, 10% glycerol/PBS was employed to mount the slides, and the results were observed under a microscope. The immunohistochemical results were independently analzyed by two people using double-blinded fashion.

### RT-qPCR

Total RNA was extracted using Trizol (15596026, Invitrogen, Car, USA), and then reverse transcribed into complementary DNA (cDNA) using a reverse transcription kit (RR047A, Takara, Japan). miRNA expression was detected according to the instructions of TaqMan® MicroRNA Assays (Applied Biosystems, Foster City, CA, USA). Reverse transcription of 10 ng Sample RNA was performed using the purposeful stem-loop primers and TaqMan® MicroRNA Reverse Transcription Kit. Using cDNA as a template, TaqMan MicroRNA Assay and TaqMan® Universal PCR Master Mix were employed for RT-qPCR. The reaction sysytem was as follow: 95 °C for 2 min, followed by 45 cycles at 95 °C for 15 s, and 60 °C for 45 s. U6 was employed as an internal reference to normalize the results. The fold changes were calculated using relative quantification (the 2−ΔΔCt method) (Table 2).

**Table 2:** Primer sequences for RT-qPCR

Primer	Sequence (5′-3′)
miR-365 (human)	Forward: 5′-GCTGTCAACGATACGCTACGT-3′
miR-365 (rat)	Forward: 5′-GCAGTAATGCCCCTAAAAATCC-3′
U6 (human)	Forward: 5′-GCTTCGGCAGCACATATACTAAAAT-3′
U6 (rat)	Forward: 5′-GCTTCGGCAGCACATATACTAAAAT-3′
	Reverse: 5′-CGCTTCACGAATTTGCGTGTCAT-3′

### Western blot assay

The total protein was extracted from tissues and cells, and the protein concentration was measured using a BCA kit (Thermo Fisher Scientific, USA). A total of 30 μg of total protein was subjected to polyacrylamide gel electrophoresis and transferred onto a PVDF membrane (Amersham, USA). The membrane was blocked with 5% skim milk powder at room temperature for 1 h and incubated at 4 °C overnight with rabbit antibodies against TLR4 (2 ug/mL, ab13556, Abcam, UK), IRF3 (1 ug/mL, ab68481, Abcam, UK), phosphorylation of IRF3 (1 mg/mL, ab76493, Abcam, UK), YAP (10 mg/mL, ab76252, Abcam, UK), CDX2 (10 mg/mL, ab76541, Abcam, UK), E-cadherin (10 mg/mL, ab40772, Abcam, UK), N-cadherin (1 mg/mL, ab18203, Abcam, UK), Bax (1: 1000, ab32503, Abcam, UK) and Bcl-2 (1: 1000, ab32124, Abcam, UK). The membrane was washed 3 times with PBST (PBS buffer containing 0.1% Tween-20), 10 min each times. Subsequently, horseradish peroxidase-labeled secondary goat anti-rabbit IgG (10 mg/mL, ab6721, Abcam, UK) was added to the membrane for incubation at room temperature for 1 h. The membrane was washed 3 times with PBST buffer for 10 min each. After scanning and development with an optical luminometer (GE Healthcare, USA), the protein band intensities were performed using Image Pro Plus 6.0 software (Media Cybernetics, USA), followed by analysis of the relative protein expression.

### Statistical analysis

SPSS 21.0 statistical software (IBM Corp. Armonk, NY, USA) was employed to analyze all experimental data. Measurement data were expressed as mean ± standard deviation. Comparison between two groups was performed using unpaired *t* test and that among multiple groups was performed using one-way analysis of variance (ANOVA). Data comparison among multiple groups at different time points was performed by two-way ANOVA. *p* < 0.05 indicated the difference was statistically significant.

## Results

### MiR-365 is poorly expressed in gastric precancerous lesions

**Fig. 1:**
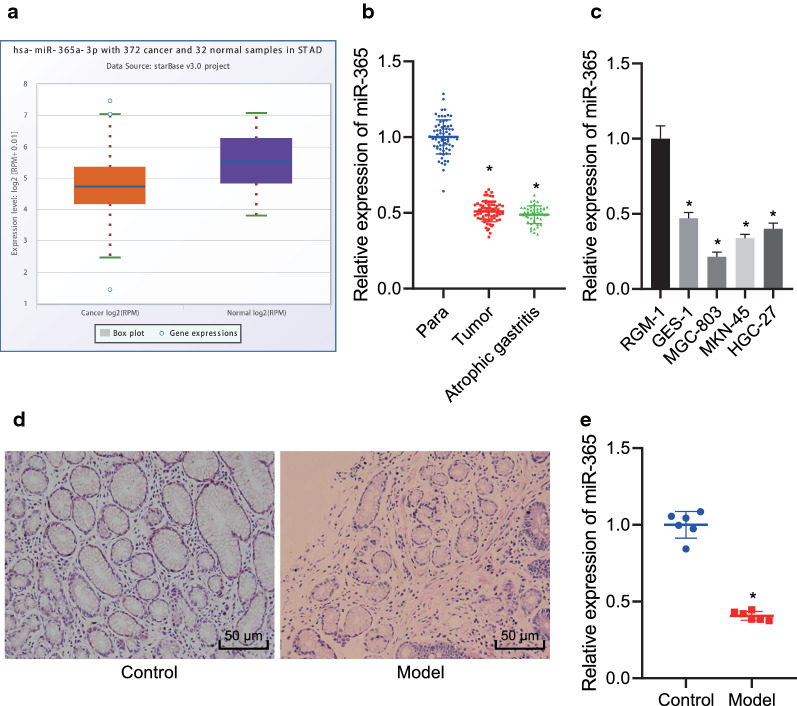
miR-365 is poorly expressed in gastric precancerous lesions. **a** The box diagram of miR-365 expression analyzed by the starBase database (https://starbase.sysu.edu.cn/). The red box on the left indicates miR-365 expression in GC samples, and the purple box on the right indicates miR-365 expression in normal samples. **b** Expression of miR-365 in GC and adjacent normal tissues of 68 GC patients and 45 atrophic gastritis patients detected by RT-qPCR; **c** Expression of miR-365 in RGM-1, GES-1, MGC-803, MKN-45, HGC-27 cell lines detected by RT-qPCR; **d** Representative images of HE staining of gastric tissue in modeled rats and control rats (magnification, ×200); **e** Expression of miR-365 in gastric tissues of normal and GC rats determined by RT-qPCR (N = 6). *, *p* < 0.05, vs. adjacent normal tissues or control rats; Measurement data (mean ± standard deviation) between two groups were compared by unpaired *t* test, and those among multiple groups were compared by one-way ANOVA. Cell experiments were repeated three times

In previous evidence, miR-365 has been demonstrated to be poorly expressed in GC [8]. The starBase database further determined that miR-365 was downregulated in GC (Fig. 1a), but miR-365 has rarely been studied in gastric precancerous lesions. Results from RT-qPCR demonstrated miR-365 mRNA expression was lower in GC and adjacent normal tissues of 68 GC patients and 45 atrophic gastritis patients (Fig. 1b) (*p* < 0.05). At the same time, RT-qPCR detected that (Fig. 1c) compared with normal human gastric mucosal epithelial cells, miR-365 expression was also diminished in induced GES-1 cells and GC cell lines MGC-803, MKN-45, and HGC-27 (*p* < 0.05). HE staining (Fig. 1d) showed that the cells in GC rats had irregular cell shape and elevated lymphocyte infiltration, indicating that the model was successfully constructed. Moreover, RT-qPCR (Fig. 1e) identified low miR-365 expression in modeled rats relative to normal rats (*p* < 0.05). These results indicated low miR-365 expression in gastric precancerous lesions.

### Overexpression of miR-365 inhibits gastric precancerous lesions

**Fig. 2:**
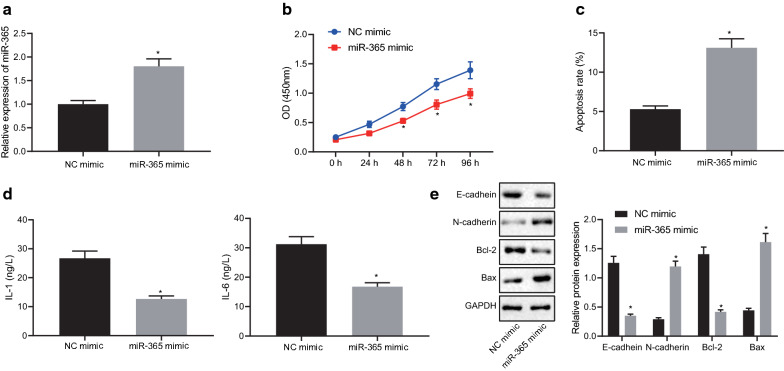
Upregulated miR-365 reverses the epithelialization of GES-1 cells induced by TNF-α. **a** miR-365 expression in TNF-α-induced GES-1 cells detected by RT-qPCR upon treatment with miR-365-mimic or NC-mimic; **b** Viability of TNF-α-induced GES-1 cells upon treatment with miR-365-mimic or NC-mimic measured by CCK-8 assay; **c** Apoptosis of TNF-α-induced GES-1 cells upon treatment with miR-365-mimic or NC-mimic measured by flow cytometry; **d** IL-1 and IL-6 contents of TNF-α-induced GES-1 cells upon treatment with miR-365-mimic or NC-mimic measured by ELISA; **e** Western blot analysis of E-cadherin, N-cadherin, Bcl-2 and Bax proteins in TNF-α-induced cells upon treatment with miR-365-mimic or NC-mimic; * *p* < 0.05, vs. NC group. Measurement data (mean ± standard deviation) between two groups were compared with unpaired *t* test, while data between multiple groups were compared with one-way ANOVA; data comparison between groups at different time points was compared with two-way ANOVA. Cell experiments were repeated three times

We further explored the effects of miR-365 on cell proliferation, apoptosis, and EMT in TNF-α-induced GES-1 cell lines. The expression of miR-365 detected by RT-qPCR presented with an elevated trend in GES-1 cell lines overexpressing miR-365 (Fig. 2a), indicating the success of transfection. In addition, as demonstrated in Fig. 2b, c, cell viability was inhibited and the apoptosis was significantly elevated after overexpression of miR-365 (*p* < 0.05). ELISA results (Fig. 2d) showed that the expression of inflammatory factors IL-1 and IL-6 was significantly reduced after miR-365 overexpression (*p* < 0.05). Further, we tested the expression of EMT-related proteins E-cadherin, N-cadherin, and apoptosis-related proteins Bax and Bcl-2, and the results depicted (Fig. 2e): E-cadhein and Bcl-2 expression were reduced but N-cadherin and Bax expression were elevated by miR-365 overexpression (*p* < 0.05). The results indicate that overexpression of miR-365 can reverse the epithelialization of GES-1 cells induced by TNF-α.

**Fig. 3:**
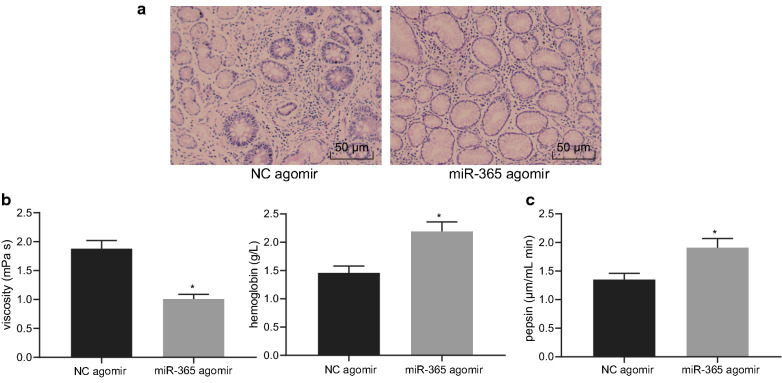
Upregulated miR-365 retards gastric precancerous lesions. **a** HE staining of gastric tissues in rats upon treatment with miR-365 agomir or NC agomir (×200); **b** Plasma analysis of rat plasma viscosity and hemoglobin content in rats upon treatment with miR-365 agomir or NC agomir (n = 6); **c** Gastric juice analysis of pepsin activity in rats upon treatment with miR-365 agomir or NC agomir (n = 6); * *p* < 0.05, vs. NC agomir group. Measurement data (mean ± standard deviation) between two groups were compared with unpaired *t* test, while data between multiple groups were compared with one-way ANOVA; data comparison between groups at different time points was compared with two-way ANOVA. Cell experiments were repeated three times

The effect of overexpression of miR-365 on gastric precancerous lesions was further verified in a rat model. HE staining (Fig. 3a) showed that the overexpression of miR-365 resulted in a more regular cell shape and reduced lymphocyte infiltration. At the same time, analysis on the plasma viscosity, hemoglobin content (Fig. 3b), and pepsin activity in gastric juice (Fig. 3c) showed that blood viscosity was decreased, while hemoglobin and pepsin activity were elevated upon miR-365 overexpression (*p* < 0.05). It was suggested that overexpression of miR-365 inhibited gastric precancerous lesions.

### miR-365 inhibits gastric precancerous lesions by inhibiting TLR4 expression

**Fig. 4:**
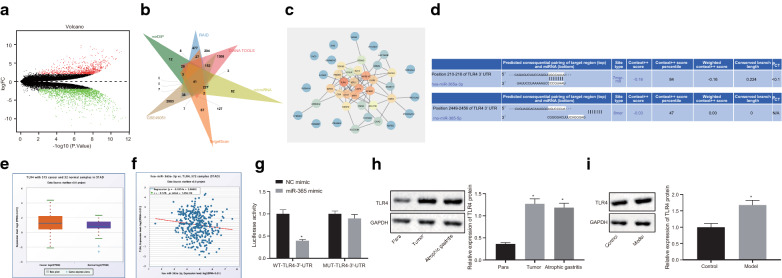
miR-365 reverses the TNF-α-induced epithelialization of GES-1 cells by inhibiting TLR4. **a** Volcano map of differentially expressed genes from the GSE49051 dataset. Red dots indicate significantly up-regulated genes, and green dots indicate significantly down-regulated genes. **b** Venn diagram of the predicted downstream genes of miR-365 by mirDIP (Minimum Score: Very High; https://ophid.utoronto.ca/mirDIP/), RAID (https://www.rna-society.org/raid2), DIANA TOOLS (miTG score > 0.5; https://diana.imis.athena-innovation.gr/DianaTools), microRNA (energy < − 10, mirsvr_score < − 0.2; https://www.microrna. org/microrna/home.do), and TargetScan (Cumulative weighted context +  + score < − 0.15; https://www.targetscan.org/vert_71/) databases and significantly differentially expressed genes from the GSE49051 dataset (with ∣logFC∣ > 2, *p* < 0.01 as differential analysis threshold). **c** PPI network of 41 intersected genes constructed by String (https://string-db.org). Cytoscape (https://cytoscape.org) was then employed to beautify the PPI network and calculate the core degree of genes. The higher the core of the gene, the reder the circle is, and the lower the core is. The lower the core of the gene, the color is bluer; **d** Binding sites between miR-365 and TLR4 in human (top) and mouse (bottom) predicted by the TargetScan; **e** A box plot of TLR4 expression analyzed by starBase, the red box on the left represents the expression in GC samples, and the purple box on the right indicates the expression in normal samples; **f** The correlation diagram of miR-365 and TLR4 expression obtained following starBase analysis, r < 0 indicates negative correlation, p-value < 0.05 indicates that the relationship is clearly acceptable; **g** The binding of miR-365 to TLR4 confirmed by dual-luciferase reporter assay, * *p* < 0.05, *vs.* cells transfected with NC mimic. **h** Western blot analysis of TLR4 protein in clinical GC tissues and adjacent normal tissues of 68 GC patients and 45 atrophic gastritis patients, * *p* < 0.05, *vs.* adjacent normal tissues; **i** Western blot analysis of TLR4 protein expression in gastric tissues of GC mice and control mice, *p < 0.05, vs. control rats; Measurement data (mean ± standard deviation) between two groups were compared with unpaired *t* test while data between multiple groups were compared with one-way ANOVA; data comparison between groups at different time points was compared with two-way ANOVA. Cell experiments were repeated three times

The aforementioned findings suggested that miR-365 could regulate gastric precancerous lesions, and we then explored the downstream mechanisms of miR-365. The databases of mirDIP, RAID, DIANA TOOLS, microRNA, and TargetScan were employed to predict the downstream genes of miR-365, and 647, 1956, 2694, 1022, and 918 genes were obtained respectively. In addition, 2502 significantly differentially expressed genes were obtained following analysis of the dataset GSE49051 (Fig. 4a). The Venn diagram revealed 41 differentially expressed downstream genes of miR-365 (Fig. 4b). A PPI network of these 41 genes was plotted and Cytoscape showed that TLR4 had the most core degree (Fig. 4c, Table 3). Online prediction using the TargetScan website illustrated that miR-365 could target TLR4 in human and mice (Fig. 4d). TLR4 has been found to be highly expressed in GC and can be employed as a biomarker for GC [9, 17]. StarBase analysis confirmed that TLR4 was highly expressed in GC and the expression of miR-365 and TLR4 was significantly negatively correlated (Fig. 4e, f). Dual-luciferase gene assay confirmed that miR-365 targeted TLR4 (Fig. 4g): miR-365 mimic had no significant effect on the luciferase activity of the MUT-TLR4-3′-UTR (*p* > 0.05), but reduced the luciferase activity of WT-TLR4-3′-UTR (*p* < 0.05), indicating that miR-365 could target TLR4. Moreover, TLR4 protein expression was significantly reduced in cells transfected with miR-365 mimic (Additional file 1: Figure S1A). Western blot analysis (Fig. 4h) showed that TLR4 was highly expressed in GC tissue and atrophic gastritis gastric tissue compared with adjacent cancer tissues (*p* < 0.05). Furthermore, a rat model was constructed and TLR4 expression in the model was detected by Western blot. As depicted in Fig. 4i, TLR4 was highly expressed in the rat model (*p* < 0.05). These results indicated that miR-365 targeted TLR4.

**Table 3:** Top 10 core genes in PPI network input genes

Rank	Gene	Degree
1	TLR4	13
2	LCP2	11
2	TNFSF10	11
4	KCNQ1	10
4	IRF8	10
6	ETS1	9
7	ITPR1	8
7	IGF2	8
7	MAFB	8
10	ANK3	7

**Fig. 5:**
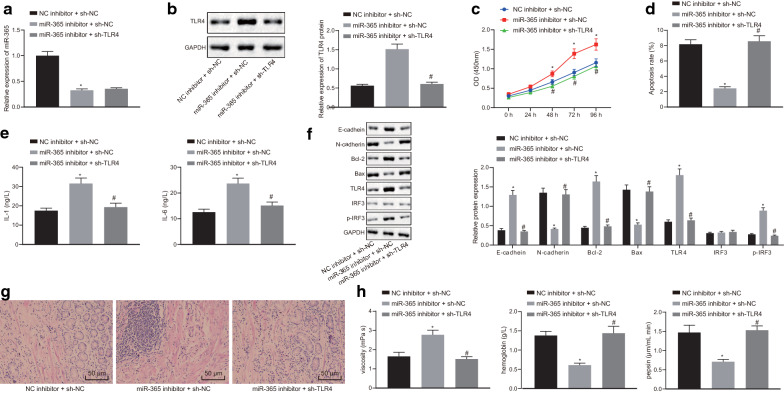
miR-365 prevents gastric precancerous lesions by inhibiting TLR4. **a** RT-qPCR analysis of miR-365 expression in GES-1 cells after transfection with miR-365 inhibitor, NC inhibitor and sh-NC. *p < 0.05, vs. cells transfected with NC inhibitor + sh-NC, # *p* < 0.05, vs. cells transfected with miR-365 inhibitor + sh-NC; **b** Western blot analysis of TLR4 protein in GES-1 cells after co-transfection with miR-365 inhibitor, NC inhibitor and sh-NC, * *p* < 0.05, vs. cells transfected with NC inhibitor + sh-NC, ^#^*p* < 0.05, vs. cells transfected with miR-365 inhibitor + sh-NC, *p* < 0.05; **c** CCK-8 assay of cell viability in GES-1 cells after co-transfection with miR-365 inhibitor, NC inhibitor and sh-NC. *p* < 0.05, vs. cells transfected with NC inhibitor + sh-NC, ^#^*p* < 0.05, vs. cells transfected with miR-365 inhibitor + sh-NC; **d** Cell apoptosis detected by flow cytometry in GES-1 cells after co-transfection with miR-365 inhibitor, NC inhibitor and sh-NC. *p < 0.05, *vs.* cells transfected with NC inhibitor + sh-NC, # *p* < 0.05, *vs.* cells transfected with miR-365 inhibitor + sh-NC; **e** IL-1 and IL-6 levels in cells in each group detected by ELISA, *p < 0.05, *vs.* cells transfected with NC inhibitor + sh-NC, # *p* < 0.05, vs. cells transfected with miR-365 inhibitor + sh-NC; **f** Western blot analysis of E-cadherin, N-cadherin, Bcl-2, Bax, TLR4, IRF3 and p-IRF3 proteins in rats after co-transfection with miR-365 antagomir, NC antagomir and sh-NC. *p < 0.05, vs. cells transfected with NC inhibitor + sh- NC, ^#^*p* < 0.05, vs*.* cells transfected with miR-365 inhibitor + sh-NC; **g** HE staining of gastric tissue lesions (×200); **h** Plasma analysis of rat plasma viscosity and hemoglobin content in rats after co-transfection with miR-365 antagomir, NC antagomir and sh-NC (n = 6). *p < 0.05, *vs.* cells transfected with NC inhibitor + sh-NC, ^#^*p* < 0.05, vs. cells transfected with miR-365 inhibitor + sh-NC; **i** Gastric juice analysis of pepsin activity in gastric juice of rats after co-transfection with miR-365 antagomir, NC antagomir and sh-NC. (n = 6), *p < 0.05, vs. cells transfected with NC inhibitor + sh-NC, ^#^*p* < 0.05, vs. cells transfected with miR-365 inhibitor + sh-NC. Measurement data (mean ± standard deviation) between two groups were compared with unpaired *t* test while data between multiple groups were compared with one-way ANOVA; data comparison between groups at different time points was compared with two-way ANOVA. Cell experiments were repeated three times

Then we investigated the effect of miR-365 on the biological characteristics of gastric precancerous lesions by regulating TLR4. We designed two sh-RNAs and detected the TLR4 expression by Western blot (Additional file 1: Figure S1B). The results showed that TLR4 expression was reduced upon sh-TLR4-1 and sh-TLR4-2 transfection, with the overexpression of TLR4-2 showing the lower TLR4 expression, so sh-TLR4-2 was selected for subsequent experiments. RT-qPCR and Western blot analysis (Fig. 5a) revealed that expression of miR-365 was diminished and the expression of TLR4 was elevated in cells transfected with 365 inhibitor + sh-NC (*p* < 0.05). Compared with the cells transfected with miR-365 inhibitor + sh-NC, the expression of miR-365 in cells transfected with miR-365 inhibitor + sh-TLR4 did not change significantly (*p* > 0.05), while TLR4 expression was diminished (*p* < 0.05). CCK-8 assay and flow cytometry (Fig. 5b, c) identified that the cell viability upon transfection with miR-365 inhibitor + sh-NC was significantly increased, and apoptosis was significantly reduced (*p* < 0.05). By contrast, cell viability upon transfection with miR-365 inhibitor + sh-TLR4 was significantly reduced, and apoptosis was significantly elevated (*p* < 0.05). Subsequently, ELISA (Fig. 5d) and Western blot analysis results (Fig. 5e) displayed that miR-365 inhibitor resulted in an elevated expression of IL-1, IL-6, E-cadherin and Bcl-2 yet diminished expression of N-cadherin and Bax (*p* < 0.05) while dual treatment with miR-365 inhibitor and sh-TLR4 presented opposite trends (*p* < 0.05). These results demonstrated that co-transfection of miR-365 inhibitor and sh-TLR4 could reverse the epithelialization of GES-1 cells induced by TNF-α.

The effects of miR-365 and TLR4 on gastric precancerous lesions were further verified in a rat model. RT-qPCR and Western blot analysis (Additional file 1: Figure S1C, D) showed that miR-365 antagomir reduced miR-365 expression and elevated TLR4 expression in a rat model. There was no significant change in miR-365 expression (*p* > 0.05), while TLR4 positive expression was diminished in response to rats with miR-365 antagomir + sh-TLR4 treatment (*p* < 0.05). HE staining (Fig. 5f) showed that rats treated with miR-365 antagomir treatment had irregular cell shape and elevated lymphocyte infiltration. However, miR-365 antagomir + sh-TLR4 treatment led to a more regular cell shape and reduced lymphocyte infiltration. Analysis of plasma viscosity and hemoglobin content (Fig. 5g), gastric juice analysis of pepsin activity in gastric juice (Fig. 5h) showed that the blood viscosity was increased, while hemoglobin content and pepsin activity were reduced following miR-365 inhibitor treatment (*p* < 0.05), which was reversed by miR-365 inhibitor + sh-TLR4 treatment (*p* < 0.05). The above-mentioned results supported that miR-365 impeded progression of gastric precancerous lesions by inhibiting TLR4.

### MiR-365/TLR4 binds to YAP in the nucleus and activates CDX2 by promoting IRF3 phosphorylation

**Fig. 6:**
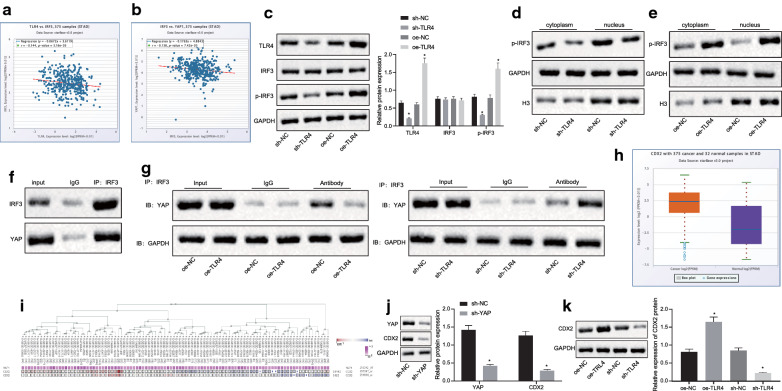
TLR4 binds to YAP in the nucleus by promoting IRF3 phosphorylation, which in turn activates CDX2. **a** Correlation diagram of TLR4 and IRF3 expression (r = − 0.144, p = 0.00516); **b** Correlation diagram of IRF3 and YAP (YAP1) expression (r = − 0.138, p = 0.00742); **c** Western blot analysis of TLR4 and phosphorylation of IRF3 in TNF-α-induced GES-1 cells transfected with sh-or oe-TLR4; **d** Western blot analysis of phosphorylation of IRF3 in TNF-α-induced GES-1 cells transfected with sh- or oe-TLR4; **e** Western blot analysis of phosphorylation of IRF3 in TNF-α-induced GES-1 cells transfected with sh- or oe-TLR4; **f** IRF3 binding to YAP in the nucleus of TNF-α-induced GES-1 cells detected by Co-IP assay; **g** IRF3 binding to YAP in cells transfected with sh- or oe-TLR4; **h** Quantitative analysis of CDX2 expression in starBase database, red box on the left indicates the expression in GC samples, purple box indicates the expression in normal samples; **i** MEM analysis (https://biit.cs.ut.ee/mem/index.cgi) of the relationship between YAP (YAP1) and CDX2 (*p* < 0.05); **j** Detection of YAP and CDX2 expression in TNF-α-induced GES-1 cells after YAP silencing; **k** Western blot analysis of CDX2 protein in TNF-α-induced GES-1 cells after over-expression of TLR4 or silencing of TLR4; *p < 0.05, vs. cells transfected with oe-NC or sh-NC. Measurement data (mean ± standard deviation) between two groups were compared with unpaired t test while data between multiple groups were compared with one-way ANOVA. Cell experiments were repeated three times

The starBase database analysis found negative correlation between TLR4 and IRF3, IRF3 and YAP1 (Fig. 6a, b). Western blot analysis showed that TLR4 and phosphorylation of IRF3 was reduced after TLR4 silencing (*p* < 0.05), and IRF3 expression showed no significantly changes (*p* > 0.05). However, TLR4 and phosphorylation of IRF3 were elevated after overexpression of TLR4 (*p* < 0.05), and IRF3 expression showed no changes (*p* > 0.05) (Fig. 6c). In addition, Western blot detected (Additional file 2: Figure S2A) that the phosphorylation of IRF3 in gastric tissue of atrophic gastritis was elevated (*p* < 0.05), and the expression of IRF3 did not change significantly (*p* > 0.05). Moreover, Additional file 2: Figure S2B showed that the expression of phosphorylation of IRF3 was elevated in the lung tissues in rat GC models (*p* < 0.05) with no significant changes in IRF3 expression (*p* > 0.05). Therefore, miR-365 might regulate the phosphorylation of IRF3 protein by targeting TLR4.

We next further verified the downstream regulatory mechanism of TLR4 in IRF3. As demonstrated in Fig. 6d, e, IRF3 phosphorylated into nucleus. After silencing TLR4, phosphorylation of IRF3 was reduced, and IRF3 expression was reduced in the nucleus (Fig. 6d). After overexpression of TLR4, the phosphorylation of IRF3 was increased, and the expression of IRF3 in the nucleus was elevated (Fig. 6e) (*p* < 0.05). Co-IP assay revealed that IRF3 in the nucleus was able to bind to YAP (Fig. 6f), and binding of IRF3 to YAP was diminished in the nucleus after TLR4 was silenced, which was abrogated by overexpression of TLR4 (Fig. 6g) (*p* < 0.05).

StarBase analysis found that CDX2 was highly expressed in GC, and MEM analysis confirmed a significant co-expression relationship between YAP (YAP1) and CDX2 (Fig. 6h, i). RT-qPCR found (Additional file 2: Figure S2C) that compared with adjacent cancer tissues, CDX2 was highly expressed in GC tissues and atrophic gastritis gastric tissues (*p* < 0.05). Moreover, CDX2 was found to be upregulated in the rat GC models (*p* < 0.05) (Additional file 2: Figure S2D). Western blot analysis (Fig. 6j) suggested that both YAP and CDX2 were downregulated in GES-1 cells upon YAP silencing (*p* < 0.05). Furthermore, CDX2 expression was elevated after overexpression of TLR4 and CDX2 expression while it was diminished after TLR4 silencing (*p* < 0.05) (Fig. 6k). The results indicated that overexpression of TLR4 promoted IRF3 phosphorylation and bound to YAP, in the nucleus thereby activating CDX2. However, silencing of TLR4 inhibited IRF3 phosphorylation and the binding to YAP in the nucleus was repressed, thereby reducing CDX2 expression.

### TLR4 inhibits YAP-mediated CDX2 transcription by inhibiting IRF3 phosphorylation and participates in gastric precancerous lesions

**Fig. 7:**
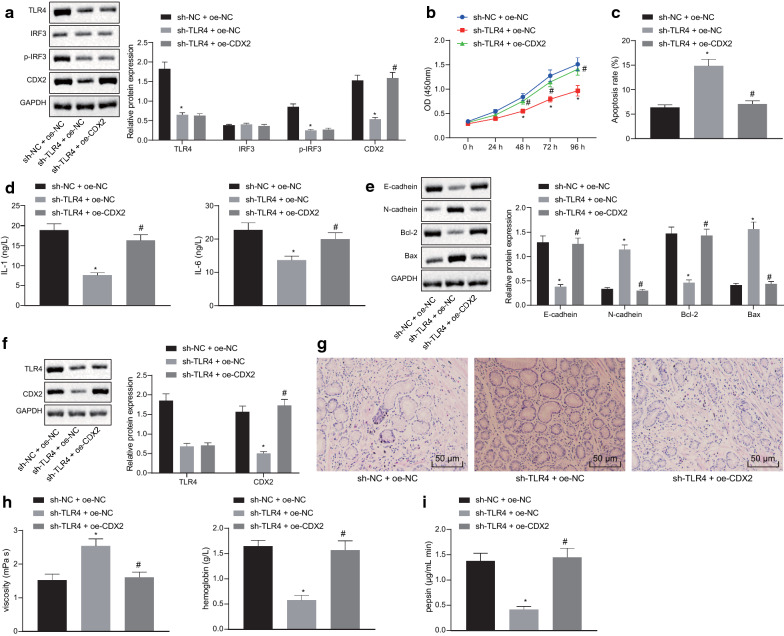
TLR4 inhibits YAP-mediated CDX2 transcription by inhibiting IRF3 phosphorylation and is involved in gastric precancerous lesions. **a** Western blot analysis of TLR4, IRF3, phosphorylation of IRF3 and CDX2 in TNF-α-induced GES-1 cells upon co-transfection with sh-TLR4 and oe-CDX2, or sh-NC or oe-NC; **b** Cell viability in each group detected by CCK-8 assay in TNF-α-induced GES-1 cells upon co-transfection with sh-TLR4 and oe-CDX2, or sh-NC or oe-NC; **c** Cell apoptosis in each group detected by flow cytometry in TNF-α-induced GES-1 cells upon co-transfection with sh-TLR4 and oe-CDX2, or sh-NC or oe-NC; **d** IL-1 and IL-6 levels in each group detected by ELISA in TNF-α-induced GES-1 cells upon co-transfection with sh-TLR4 and oe-CDX2, or sh-NC or oe-NC; **e** Western blot analysis of E-cadherin, N-cadherin, Bcl-2, Bax, TLR4, IRF3 and phosphorylation of IRF3 in TNF-α-induced GES-1 cells upon co-transfection with sh-TLR4 and oe-CDX2, or sh-NC or oe-NC; **f** Western blot analysis of TLR4 and CDX2; **g** HE staining of gastric tissue lesions of rats upon co-transfection with sh-TLR4 and oe-CDX2, or sh-NC or oe-NC (×200); **h** Plasma viscosity and hemoglobin content of rats in plasma analysis upon co-transfection with sh-TLR4 and oe-CDX2, or sh-NC or oe-NC (n = 6); **i** Pepsin content of gastric juice in gastric juice analysis of rats upon co-transfection with sh-TLR4 and oe-CDX2, or sh-NC or oe-NC (n = 6); *p < 0.05, vs. rats treated with sh-NC + oe-NC, ^#^*p* < 0.05, vs. rats treated with sh-TLR4 + oe-NC. Measurement data (mean ± standard deviation) between two groups were compared with unpaired *t* test while data between multiple groups were compared with one-way ANOVA; data comparison between groups at different time points were compared with two-way ANOVA. Cell experiments were repeated three times

In the foregoing, we confirmed that TLR4 bound to YAP in the nucleus by promoting IRF3 phosphorylation, and then activated CDX2. Thereafter, we further explored the effect of TLR4 on the biological characteristics of gastric precancerous lesions through CDX2. Western blot analysis (Fig. 7a) clarified that expression of TLR4, phosphorylation of IRF3 and CDX2 were reduced in sh-TLR4-transfected cells (*p* < 0.05), and IRF3 expression was not significantly changed (*p* > 0.05). Co-transfection with sh-TLR4 and oe-CDX2 led to no changes in expression of TLR4, IRF3 and phosphorylation of IRF3 (*p* > 0.05), and CDX2 expression was elevated (*p* < 0.05). Cell viability and apoptosis were detected by CCK-8 assay and flow cytometry (Fig. 7b, c), and the results demonstrated that the cell viability upon sh-TLR4 + oe-NC transfection was significantly reduced, and apoptosis was significantly elevated (*p* < 0.05) while sh-TLR4 + oe-CDX2 transfection showed a reverse trend (*p* < 0.05). Subsequently, ELISA (Fig. 7d) and Western blot analysis results (Fig. 7e) displayed that the expression of IL-1, IL-6, E-cadherin and Bcl-2 in the sh-TLR4 + oe-NC-transfected cells was significantly reduced, but the expression of N-cadherin and Bax was elevated (*p* < 0.05) while sh-TLR4 + oe-CDX2-transfected cells showed opposite trends (*p* < 0.05).

The effect of co-transfection of sh-TLR4 and oe-CDX2 on gastric precancerous lesions was further verified in a rat model. The expression of TLR4 and CDX2 was detected by Western blot (Fig. 7f). The results indicated that the expression of TLR4 and CDX2 was reduced in sh-TLR4 + oe-NC-treated rats (*p* < 0.05) while the expression of TLR4 in sh-TLR4 + oe-CDX2 was not significantly changed (*p* > 0.05), and CDX2 expression was elevated (*p* < 0.05). HE staining (Fig. 7g) presented that the cell shape of sh-TLR4 + oe-NC-treated rats was regular, and lymphocyte infiltration was reduced while the sh-NC + oe-NC-treated rats had irregular cell shape and elevated lymphocyte infiltration. Analysis of plasma viscosity and hemoglobin content (Fig. 7h) and gastric juice analysis of pepsin content in gastric juice (Fig. 7I) found that blood viscosity of sh-TLR4 + oe-NC-treated rats was reduced, while the contents of hemoglobin and pepsin elevated (*p* < 0.05), which was rescued by co-treatment with sh-TLR4 and oe-CDX2 (*p* < 0.05). The collected data demonstrated that TLR4 was involved in gastric precancerous lesions by inhibiting IRF3 phosphorylation and thus inhibiting YAP-mediated CDX2 transcription.

**Fig. 8:**
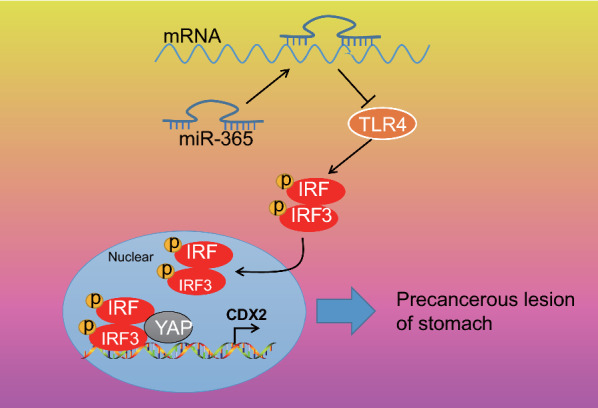
A molecular mechanism diagram depicting the role of miR-365 in gastric precancerous lesions. miR-365 targets TLR4 and inhibits its expression. Overexpression of TLR4 promotes the phosphorylation of IRF3 into the nucleus and binds to YAP, thereby activating CDX2 and promoting gastric precancerous lesions

## Discussion

GC remains a prevalent disease worldwide with a poor prognosis [18]. GC is the second leading cause of cancer death after lung cancer [19]. The prognosis is poor, with an average 5-year survival rate of less than 20%, mainly due to the late diagnosis [20]. Therefore, early diagnosis of GC is particularly important in the diagnosis. Studying the mechanism of gastric precancerous lesions can help develop early clinical detection and diagnosis of GC. The survival rate of GC patients provides a reliable theoretical basis. In this study, in vitro and in vivo experiment demonstrated that overexpression of miR-365 could potentially reduce IRF3 phosphorylation and YAP-mediated CDX2 transcription, thereby alleviating gastric precancerous lesions.

The current study demonstrated that miR-365 showed low expression in gastric tissues and different GC cells in patients with atrophic gastritis. It is suggested that miR-365 may have a certain indicator role in gastric precancerous lesions, but no related mechanism has been studied. Consistently, previous studies also note that miR-365 is poorly expressed in gastric cancer and its upregulation can impede gastric tumorigenesis [8]. Of the deletion and mutation of the miR-365 promoter, transcription factors Sp1 and NF-κB are essential for miR-365 transcription regulation [21]. The TLR4/NF-κB signaling pathway plays multiple roles in coronary microembolization [22], gastroesophageal reflux disease [23], and diabetic nephropathy [24]. In GC, TLR4 is amplified in GC and can be employed as a biomarker for GC [9]. Importantly, our work indicated that miR-365 can target TLR4 and inhibit its expression when retarding GC development. miRNAs might suppress EMT which contributes to metastasis during cancer progression [25]. EMT is involved in tumor cell migration and intravasation to the blood in various tumors, but its process predominantly depends on different cancers and surrounding stimuli such as transcription factors and miRNA [26]. miR-365 in present study was indicated to downregulate E-cadhein and Bcl-2 expression, EMT-related proteins. But its potential effect on GC metastasis requires further experiments. Similarly, a recent report also demonstrated that miR-365 could repress EMT in lung adenocarcinoma through targeting ETS1 [27].

In recent years, Toll-like receptors (TLRs) have been highlighted for their role in immune reaction, where components of microorganisms such as lipopolysaccharides (LPS) are recognized by TLRs, thereby activating tumor cells [28]. TLR4 is an important LPS receptor in gastric epithelial cell signaling transduction and promotes GC progression [29]. TLR4 mediates IRF3 activation could induce the transcription of inflammatory factors for macrophage activation in GC [11]. The absence of TLR4 also leads to reduced release of phosphorylated interferon-regulated transcription factor (p-IRF3) and interferon (IFN-β) [30]. Interestingly, the current study unraveled molecular mechanism that overexpression of TLR4 promotes IRF3 phosphorylation to bind more YAP, activating CDX2. IRF3 was previously implicated as an agonist of YAP and IRF3 was positively correlated with YAP in gastric cancer [12]. Amlexanox, a drug for inflammation, inhibits GC growth in a YAP-dependent manner. In addition, CDX2 contributes to gastric intestinal metaplasia, and is active in GC [31]. But the interaction among CDX2, TLR4, YAP and IRF3 has rarely been discussed in the disorder and still requires more evidence to identify the direct or indirect relation between these molecules and miR-365.

## Conclusion

In summary, we report for the first time that in human gastric precancerous lesions, low expression of miR-365 is associated with the downstream target gene TLR4, and that TLR4 binds to YAP in the nucleus by promoting IRF3 phosphorylation, and then activates CDX2 (Fig. 8). These findings suggest that miR-365-TLR4 -phosphorylation of IRF3-YAP-CDX2 may be a therapeutic target for gastric precancerous lesions. Although our research has identified possible mechanisms of miR-365-TLR4-IRF3-YAP-CDX2 in gastric precancerous lesions, we have not revealed in more detail the more mechanisms involved as precancerous lesions, and occurrence is often multifactorial, so further research on gastric precancerous lesions has more mechanisms that have not yet been discovered, and more research attention is yet to be paid.

## Supplementary information

**Additional file 1: Figure S1.** Regulation of miR-365 on TLR4 expression. A: Western blot analysis of TLR4 protein in TNF-α-induced GES-1 cells transfected with miR-365 mimic; B: sh-RNA screening in TNF-α-induced GES-1 cells; C: miR-365 expression detected by RT-qPCR in rats upon co-transfection with miR-365 antagomir, sh-TLR4, NC antagomir, or sh-NC; D: Western blot analysis of TLR4 protein in rats upon co-transfection with miR-365 antagomir, sh-TLR4, NC antagomir; * *p* < 0.05, *vs.* rats treated with NC antagomir + sh-NC, Measurement data (mean ± standard deviation) between two groups were compared with unpaired *t* test while data between multiple groups were compared with one-way ANOVA. Cell experiments were repeated three times

**Additional file 2: Figure S2.** TLR4 binds to YAP in the nucleus by promoting IRF3 phosphorylation, and then activates CDX2. A: Western blot analysis of IRF3 and p-IRF3 in GC and adjacent normal tissues in clinical samples; B: Western blot analysis of IRF3 and p-IRF3 in GC and adjacent normal tissues of rats (n = 6); C: The expression of CDX2 in GC and adjacent normal tissues of clinical samples detected by RT-qPCR; D, The expression of CDX2 in GC and adjacent normal tissues of rats detected by RT-qPCR. Measurement data (mean ± standard deviation) between two groups were compared with unpaired *t* test while data between multiple groups were compared with one-way ANOVA. Cell experiments were repeated three times.

## Data Availability

The datasets generated/analysed during the current study are available.

## Abbreviations

GC: Gastric carcinoma
miRNAs: MicroRNAs
IRF3: Interferon regulatory factor 3
TNF-α: Tumor necrosis factor-alpha
GES-1: Gastric mucosal epithelial cells
MNNG: *N*-Methyl*N*-nitro-*N*-nitroso-guanidine
TMB: The tetramethylbenzidine
OD: Optical density
WT: Wild type
cDNA: Complementary DNA
ANOVA: Analysis of variance
TLRs: Toll-like receptors
LPS: Lipopolysaccharides
p-IRF3: Phosphorylated interferon-regulated transcription factor
